# Supplementary material for: Food Environments and Hepatocellular Carcinoma Incidence
Source: Int J Environ Res Public Health. 2021 May 27;18(11):5740. doi: 10.3390/ijerph18115740 (PMC8198353; doi:10.3390/ijerph18115740)
Supplement: Supplementary file 1 [file ijerph-18-05740-s001.zip › ijerph-1191686-supplementary.pdf]

Supplemental Table S1. Associations between mRFEI exposure and HCC incidence stratified by age at diagnosis, year at diagnosis, race/ethnicity, and region (SEER 2000-2016)

		**Fully adjusted‡**	
**mRFEI† exposure**	**N cases**	**IRR (95% CI)**	**p for interaction**
Age at diagnosis			<0.01
<65 years	53,123	1.01 (0.98, 1.05)	
≥65 years	37,455	0.96 (0.93, 0.99)	
Race/ethnicity			<0.01
Non-Hispanic white	45,404	1.03 (0.99, 1.07)	
Non-Hispanic black	12,841	0.99 (0.92, 1.05)	
Hispanic	17,804	0.95 (0.89, 1.01)	
Non-Hispanic Asian or Pacific Islander	13,713	0.94 (0.88, 0.99)	
Year at diagnosis			0.01
2000-2007	32,362	0.99 (0.95, 1.02)	
2008-2016	58,216	0.99 (0.85, 1.01)	
Region			<0.01
West	41,728	0.99 (0.92, 1.07)	
Midwest	4,835	0.94 (0.88, 1.01)	
South	12,387	0.95 (0.93, 1.04)	
Northeast	31,628	0.84 (0.54, 1.31)	

†mRFEI (per IQR increase of 9.3)

‡Adjusted for age at diagnosis, sex, race/ethnicity, year, and SEER registry, and county-level measures of urbanicity, median household income, percentage with Bachelor's degree or higher, percentage unemployed, percentage of individuals below the poverty level, percentage of foreign born, and prevalence of alcohol consumption, smoking, obesity, and diabetes
